# Adding another piece to the retinoblastoma puzzle

**DOI:** 10.1038/cddis.2015.317

**Published:** 2015-10-29

**Authors:** M K E Blixt, S Shirazi Fard, C All-Ericsson, F Hallböök

**Affiliations:** 1 Department of Neuroscience, Biomedical Center, Uppsala University, Uppsala, Sweden; 2 S:t Eriks ögonsjukhus, Department of Clinical Neuroscience, Karolinska Institutet, Stockholm, Sweden

The transcriptional cofactor *retinoblastoma 1* (*RB1*) was the first gene to be identified as a tumour suppressor, and was later found to be important for restricting the G1-S phase transition of the cell cycle. *RB1* mutations in the germline confer predisposition to retinoblastoma, however sporadic mutations are more frequent (70%). In addition, in about 10% of the cases,^1^ *RB1* mutations are not found indicating other causative mutations or events.^2^ Retinoblastoma is a rare childhood cancer, with a reported incidence of 1 in 15–18 000 live births. Since the discovery of *RB1* mutations, much effort has been put into finding the ‘cell-of-origin' for retinoblastoma and elucidating the underlying mechanisms. Following *RB1* knockdown in human foetal retinal cells, proliferation of cone photoreceptor precursor cells was induced, and when xenografted, Rb-depleted cone precursors formed tumours.^3^ This shows that postmitotic human cone precursors are sensitive to Rb depletion. However, in a study performed on mice, it was reported that the retinal horizontal cells are able to re-enter the cell cycle, expand clonally and form metastatic tumours.^4^ Horizontal cells may therefore also be a ‘cell-of-origin' for retinoblastoma. This poses the question why certain cell types are more prone to become malignant following loss-of-function of *RB1*.

Both the photoreceptors and the horizontal cells are among the first retinal cells to be generated during development and they are derived from the same multipotent progenitor.^5^ Whether the ‘cell-of-origin' for retinoblastoma is a photoreceptor or a horizontal cell is maybe less important from a mechanistic perspective. However, establishing the molecular pathways that distinguish the properties of these cells from other retinal cells is crucial. Studies performed on the horizontal cells in the chicken retina have revealed intriguing results that may aid our understanding why these cells have a propensity for neoplastic transformation. A subtype of horizontal progenitor cells, those expressing Lim1 (Lim homeobox protein 1; Lim1+), undergo an S-phase that is not followed by any mitosis and subsequently becomes aneuploid.^6^ These cells neither activate the DNA damage response pathway nor undergo apoptosis.^7^ In addition, these cells are able to enter mitosis even in the presence of DNA damage, despite having a functional p53-p21 system.^8^ p53 is a tumour suppressor protein and transcription factor that constitutes a central component of the DNA damage response pathway and arrests the cell cycle by activation of the cell cycle inhibitor p21.^9^ The transcription factor Zac1 (zinc finger protein that regulates apoptosis and cell cycle arrest) interacts with and enhances the activity of p53.^10^ Zac1 was first isolated by Spengler *et al.*,^11^ and has later been identified in several tumours as a tumour suppressor gene based on its ability to control cell cycle progression and apoptosis.

In a new study by Shirazi Fard *et al.*^12^ the effect of Zac1 was investigated in the chicken embryonic retina. A gain-of-function assay was used in which a mouse Zac1 (mZac1) DNA sequence, previously used for studies in both the mouse and frog retina,^13, 14^ was expressed. Shirazi Fard *et al.* show that overexpression of mZac1 in the chicken retina results in induced expression of the cell cycle inhibitor p21. It was also established that the increase was regulated by the tumour suppressor protein p53.^12^ In another study performed in the mouse retina, removal of Zac1 resulted in an increased number of cells. Zac1 was therefore suggested to be a negative regulator of cell number and retina size, which is consistent with a function as a tumour suppressor gene.^13^ This conclusion was further supported by the results gained from the mZac1 overexpression study performed in the chicken, where a reduction in the number of cells entering S-phase and G2/M-phase was observed, confirming that Zac1 promotes cell cycle arrest and/or exit. In addition, Zac1-induced p53-activity also triggers apoptosis in the cells that overexpress mZac1 (Figure 1).^12^ These results are consistent with previous findings showing that Zac1 has the ability to inhibit cell growth and induce apoptosis.^11^

Interestingly, not all of the chicken retinal progenitor cells investigated by Shirazi Fard *et al.* arrested their cell cycles following overexpression of mZac1. The number of Lim1+ horizontal progenitor cells was not affected and this indicates that these cells are able to withstand the effect of p53 activation. In addition, the Lim1+ horizontal progenitor cells were able to enter both the S- and the G2/M-phases despite overexpression of mZac1 (Figure 1). These newly presented findings support previous studies showing that the Lim1+ horizontal progenitor cells progress through their final cell cycle and enter mitosis even in the presence of DNA damage,^8^ despite having an active DNA damage response pathway.^7^

The ability of Zac1 to function as a coactivator is dependent on association to a functional p53 protein.^10^ The Hallböök group has previously demonstrated that the horizontal cells may trigger a functional p53 response.^8^ However, there seems to be a limitation in the ability of the Lim1+ horizontal cells to execute the response following DNA damage. This discrepancy in p53 regulation might also influence the ability of Zac1 to interact with p53 in the horizontal cells.

In conclusion, the horizontal cells seem to have an atypical regulation or execution of their p53-p21 system not only after DNA damage but also when it comes to the modulators of p53, as shown by Shirazi Fard *et al.* The inability of Zac1 to arrest the cell cycle of the horizontal progenitor cells further strengthens the notion that the horizontal cells are less sensitive to signals that regulate cell cycle progression. These findings take us one step further in the quest to understand how and why certain cells are more prone to form tumours.

## Figures and Tables

**Figure 1:**
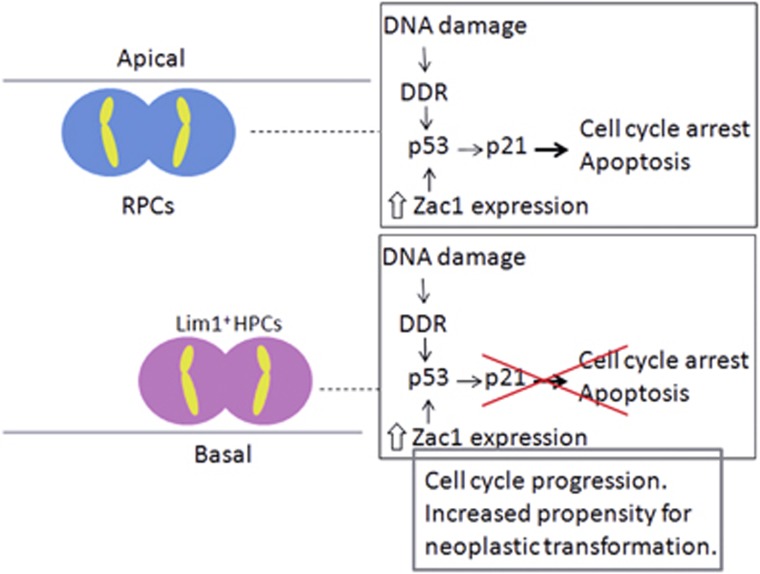
Resistance to the p53-coactivator Zac1 and DNA damage in chicken horizontal progenitor cells. Overexpression of mZac1 in the embryonic chicken retina induce p53-dependent upregulation of p21 which leads to arrest of the cell cycle and/or apoptosis in a majority of the retinal progenitor cells. The horizontal progenitor cells resist the overexpression of Zac1 and progress through the cell cycle. This deviating behaviour during their terminal mitosis may be another piece of the puzzle for understanding why certain retinal cells have a propensity for neoplastic transformation and form retinoblastoma. DDR, DNA damage response pathway; HPCs, horizontal progenitor cells; p53, tumour suppressor gene p53; p21, cyclin-dependent kinase inhibitor 1 (CIP1/warf1); RPCs, retinal progenitor cells. Apical (ventricular) and basal (vitreal) side of the retinal neuroepithelium
